# Therapeutic Strategies Against COVID-19 and Structural Characterization of SARS-CoV-2: A Review

**DOI:** 10.3389/fmicb.2020.01723

**Published:** 2020-07-14

**Authors:** Gi Uk Jeong, Hanra Song, Gun Young Yoon, Doyoun Kim, Young-Chan Kwon

**Affiliations:** ^1^Center for Convergence for Emerging Virus Infection, Korea Research Institute of Chemical Technology (KRICT), Daejeon, South Korea; ^2^Division of Therapeutics and Biotechnology, KRICT, Daejeon, South Korea

**Keywords:** COVID-19, SARS-CoV-2, 2019-nCoV, antiviral agents, therapeutic strategies, crystal structure

## Abstract

The novel coronavirus, SARS-CoV-2, or 2019-nCoV, which originated in Wuhan, Hubei province, China in December 2019, is a grave threat to public health worldwide. A total of 3,672,238 confirmed cases of coronavirus disease 2019 (COVID-19) and 254,045 deaths were reported globally up to May 7, 2020. However, approved antiviral agents for the treatment of patients with COVID-19 remain unavailable. Drug repurposing of approved antivirals against other viruses such as HIV or Ebola virus is one of the most practical strategies to develop effective antiviral agents against SARS-CoV-2. A combination of repurposed drugs can improve the efficacy of treatment, and structure-based drug design can be employed to specifically target SARS-CoV-2. This review discusses therapeutic strategies using promising antiviral agents against SARS-CoV-2. In addition, structural characterization of potentially therapeutic viral or host cellular targets associated with COVID-19 have been discussed to refine structure-based drug design strategies.

## Introduction

In late December 2019, a newly identified coronavirus strain capable of crossing the species barrier and infecting humans was first reported in Wuhan, Hubei province, China, and was provisionally termed 2019 novel coronavirus (Lu et al., 2020; Zhu et al., 2020). This novel virus was later designated as severe acute respiratory syndrome coronavirus 2 (SARS-CoV-2), owing to its genetic similarity with other coronavirus strains (Gorbalenya et al., 2020). It is known to cause coronavirus disease 2019 (COVID-19), characterized by influenza-like mild or moderate respiratory symptoms including dry cough, fever, headache, and pneumonia, as well as severe lung injury and multi-organ failure, which eventually lead to death (Chen et al., 2020; Huang C. et al., 2020). The World Health Organization (WHO) officially declared COVID-19 as a pandemic on March 11, 2020 due to the rapid global dissemination of SARS-CoV-2.

According to the WHO, a total of 3,672,238 confirmed cases of COVID-19 and 254,045 deaths were recorded up to May 7, 2020 in over 200 countries. Moreover, effective antiviral therapeutic agents or vaccines are not yet available for COVID-19. The repurposing of existing drugs designed for other viruses is the most practical strategy to treat patients with COVID-19 because they have already been tested for their safety. Although *de novo* development of antivirals is a time-, cost-, and effort-intensive endeavor, it is important to generate specific antivirals for SARS-CoV-2 that directly target the viral or host proviral factors (Cascella et al., 2020; Senanayake, 2020).

With increasing structural data of key proteins in both SARS-CoV-2 and the host, such as the spike glycoprotein (*S*), the main protease (M^pro^), RNA-dependent RNA polymerase (RdRp), and human angiotensin-converting enzyme 2 (hACE2), the structure-based design of new drugs has emerged as the most promising antiviral strategy. In this review, we have summarized the promising therapeutic potential of pre-existing drugs against COVID-19. In addition, the structural characterization of potentially therapeutic viral or host cellular targets associated with COVID-19 have been discussed to refine structure-based drug design strategies.

## SARS-CoV-2

SARS-CoV-2 is an enveloped, positive-sense, single-stranded RNA virus and belongs to the genus *Betacoronavirus*, which also includes SARS-CoV and MERS-CoV (Andersen et al., 2020; Lu et al., 2020; Zhu et al., 2020). The genome sequence of SARS-CoV-2 is more closely related to that of SARS-CoV (79% identity) than with that of MERS-CoV (~50%) (Lu et al., 2020). Notably, the S protein of SARS-CoV-2 and SARS-CoV are highly homologous with 76.5% amino acid sequence identity (Xu et al., 2020). Consequently, SARS-CoV-2 and SARS-CoV are believed to bind to the same host cell entry receptor hACE2 (Hoffmann et al., 2020; Zhou et al., 2020) instead of human dipeptidyl peptidase 4 (hDPP4), which is used by MERS-CoV (Raj et al., 2013).

**Figure 1:**
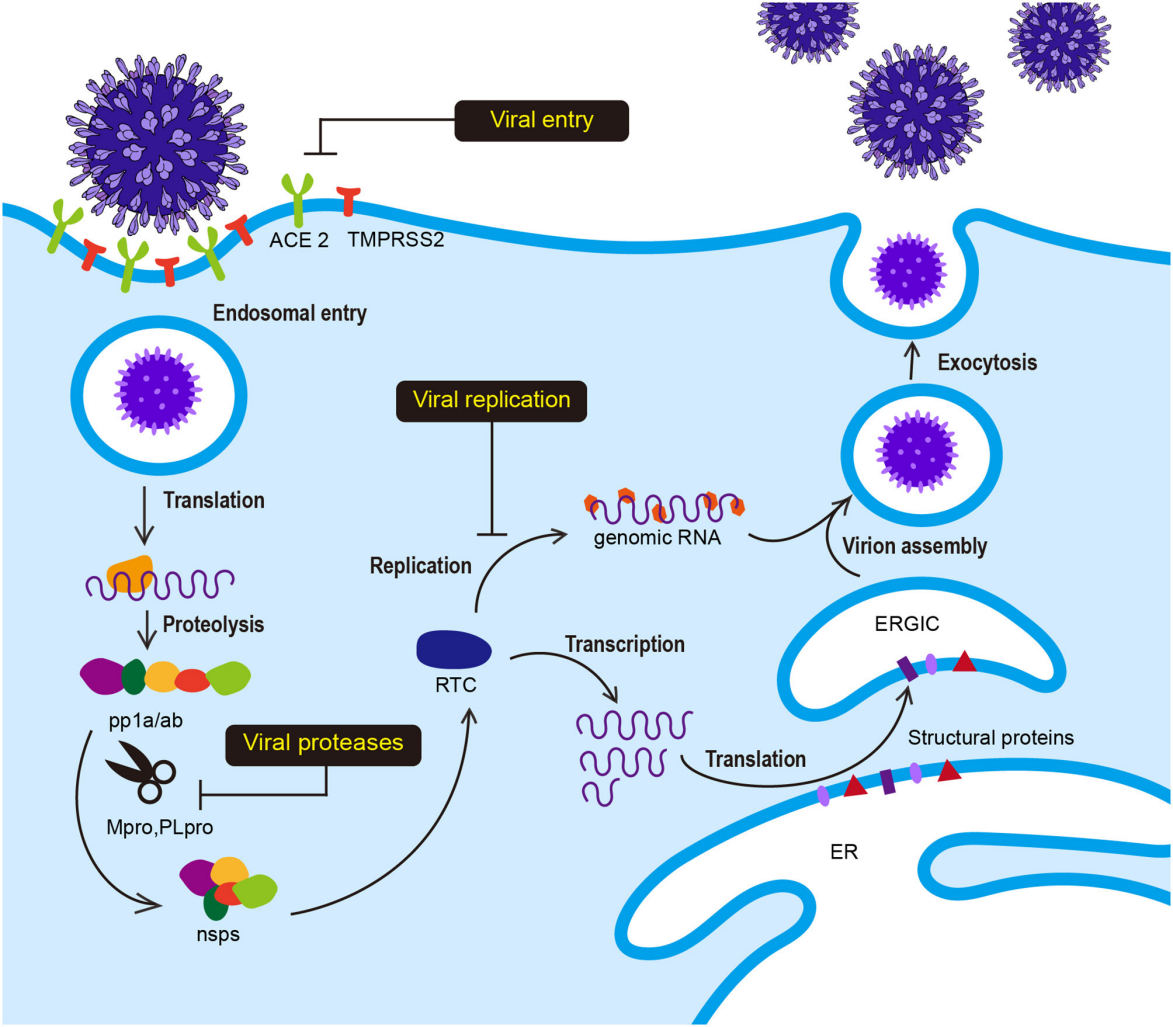
Viral life cycle of SARS-CoV-2. Interaction between the S protein of SARS-CoV-2 and hACE2 initiates SARS-CoV-2 infection. Following receptor binding, the virus enters the cell by acid-dependent proteolytic cleavage of the S protein by TMPRSS2 or other proteases. Upon fusion of the viral and host cell membranes, viral genomic RNA is released in the cytoplasm. The viral RNA initiates translation of co-terminal polyproteins (pp1a/ab) by−1 frameshifting. These polyproteins are subsequently cleaved into nonstructural proteins (nsps) by M^pro^ and PL^pro^. Several nsp proteins interact with nsp12 (RdRp) to form the replicase-transcriptase complex (RTC), which is responsible for the synthesis of full-length viral genome (replication) and sub-genomic RNAs (transcription). The viral structural proteins are expressed and translocated into the endoplasmic reticulum (ER). The nucleocapsid (*N*) protein-encapsidated genomic RNA translocates with the structural proteins into the ER-Golgi intermediate compartment (ERGIC) for virion assembly. The newly synthesized virions are budded through the cell membrane and exocytosed.

SARS-CoV-2 has club-like spikes on its surface and a distinct replication strategy analogous to other coronaviruses. The life cycle and replication of SARS-CoV-2 is shown in Figure 1. Viral infection is initiated by the interaction between the S protein and hACE2, followed by subsequent endocytosis or membrane fusion. The S protein comprises two subunits: S1 and S2. The S1 subunit contains the receptor binding domain (RBD) and binds to N-terminal hACE2, while the S2 subunit mediates virus-host membrane fusion. S proteins are cleaved by the host cell furin protease and transmembrane serine protease 2 (TMPRSS2) at the S1/S2 boundary and the S2′ position. Proteolytic cleavage at the S1/S2 boundary is thought to promote TMPRSS2-dependent entry into the target cells (Belouzard et al., 2009; Hoffmann et al., 2020; Walls et al., 2020). After the release of the viral polycistronic RNA into the cytoplasm, the replicase gene comprising open reading frames (ORFs) 1a and 1ab is directly translated into either replicase polyprotein pp1a (~450 kDa, nsp1-11) or pp1ab (~750 kDa, nsp1-16) by a ribosomal−1 frameshift near the 3′-end of ORF 1a and autoproteolytically cleaved into 16 non-structural proteins (nsp1-16) by two ORF1a-encoded protease domains (Brierley et al., 1989; Herold et al., 1993; Thiel et al., 2001, 2003; Harcourt et al., 2004; Prentice et al., 2004; Ziebuhr, 2004). Furthermore, the main protease M^pro^ (also called 3CL^pro^) and papain-like protease (PL^pro^) participate in this extensive proteolytic cleavage. The large pp1ab polyprotein has no <11 conserved cleavage sites that are mediated by M^pro^, which cleaves at Leu-Gln↓(Ser, Ala, Gly) (arrow indicates the cleavage site) (Ziebuhr et al., 2000; Hegyi and Ziebuhr, 2002). Positive-strand RNA viruses usually form a cytoplasmic enzyme complex called replicase-transcriptase complex (RTC) that can mediate the synthesis of the full-length genome (replication) or discontinuous mRNAs (transcription) (Gorbalenya et al., 2006; Pasternak et al., 2006; Sawicki et al., 2007). Structural and accessory proteins are subsequently translated from these transcripts, and new viruses assemble by budding into the lumen of the endoplasmic reticulum-Golgi intermediate compartment (ERGIC) and are eventually secreted (Klumperman et al., 1994; Hogue and Machamer, 2008).

## Antiviral Strategies Against SARS-CoV-2

Antivirals can be broadly divided into two categories: direct-acting antivirals (DAA) and indirect-acting antivirals (IAA). DAAs directly target specific viral components, such as viral polymerase, or steps in the viral life cycle without affecting other host cellular processes. The development of DAAs can facilitate the treatment of patients with COVID-19. In contrast, IAAs target host proviral factors and indirectly inhibit viral infection or replication by impeding the function or interaction of these factors. IAAs have an advantage over DAAs because they are not susceptible to viral mutations, which are frequently found in RNA viruses. However, IAAs can alter the host cellular system and are not considered safe. Therefore, DAAs targeting viral entry, proteases, and replication can serve as effective antivirals owing to their enhanced safety features. Drug repurposing of pre-existing antiviral agents is considered one of the most practical strategies because there is no available approved antiviral drug or vaccine for COVID-19. Furthermore, the *de novo* development of drugs typically requires over $1 billion USD and 10–17 years (Cascella et al., 2020; Senanayake, 2020). Drug repurposing of several approved antivirals against COVID-19 has progressed into clinical trials (Table 1). However, there is a potential risk of drug-resistant mutations with the use of DAA. A combination of repurposed drugs can reduce the time, cost of treatment, and risk of drug-resistance, and increase therapeutic efficacy to facilitate progression into clinical trials (Cheng et al., 2019). Moreover, due to the existence of crystal structures of viral and host cellular proteins associated with SARS-CoV-2, such as S protein, M^pro^, RdRp, and hACE2, structure-based drug design can be performed to develop more effective drugs with reduced off-target toxicity (Schomburg and Rarey, 2014).

**Table 1:** Current potential antiviral agents against SARS-CoV-2.

	**Target**	**Antiviral agent**	**Type**	**Status**	**References**
Viral entry	ACE2	HrsACE2	Recombinant protein	–	Monteil et al., 2020
	S protein	CR3022	Neutralizing antibody	–	Yuan et al., 2020
	Endocytosis	Umifenovir (Arbidol)	Membrane fusion inhibitor	Phase 4	Zumla et al., 2016; Sanders et al., 2020
	Endocytosis	EK1C4	Pan-coronavirus fusion inhibitor	–	Xia et al., 2020
	TMPRSS2	Camostat mesilate	Serine protease inhibitor	Phase 4	Hoffmann et al., 2020; Sanders et al., 2020
	TMPRSS2	Nafamostat	Serine protease inhibitor	Phase 2	Zumla et al., 2016; Bittmann et al., 2020
	TMPRSS2	Bromhexine hydrochloride	Mucolytic drug	–	Sonawane et al., 2020
	TMPRSS2	PAI-1	Serine protease inhibitor	–	Jankun, 2020
	Virus/Cell fusion	Chloroquine	Drug for autoimmune disease	Phase 4	Wang M. et al., 2020
Viral proteases	M^pro^	Lopinavir/Ritonavir	Antiviral	Phase 4	Harrison, 2020; Sanders et al., 2020
	M^pro^	Darunavir/covicistat	Antiviral	Phase 3	
	M^pro^	ASC09F/Oseltamivir	Antiviral	Phase 3	Lv et al., 2015; Zumla et al., 2016
	M^pro^	Nelfinavir	Antiviral	–	Yamamoto et al., 2020
	M^pro^	Baicalein	Antibacterial	–	Liu et al., 2020
	M^pro^	a-ketoamide	Antiviral	–	Zhang et al., 2020b
	M^pro^	Ebselen	Anti-inflammatory	–	Jin et al., 2020
	M^pro^	Disulfiram	Drug for chronic alcoholism	-	
	M^pro^	Tideglusib	Kinase inhibitor	–	
	M^pro^	Carmofur	Anticancer	–	
	M^pro^	Shikonin	Natural product	–	
	M^pro^	PX-12	Anticancer	–	
	M^pro^	Compound 11a, 11b	Antiviral	–	Dai et al., 2020
Viral replication	RdRp	Remdesivir	Antiviral	Phase 3	Zumla et al., 2016; Sanders et al., 2020; Wang M. et al., 2020
	RdRp	Ribavirin	Antiviral	Phase 2	
	RdRp	Favipiravir	Antiviral	Phase 4	
	RdRp	Galidesivir	Antiviral	Phase 1	Elfiky, 2020
	RdRp	EIDD-1931	Antiviral	–	Sheahan et al., 2020
	RdRp	EIDD-2801	Antiviral	–	
	RdRp	Tenofovir disoproxil	Antiviral	Phase 3	Elfiky, 2020

### Viral Entry

**Figure 2:**
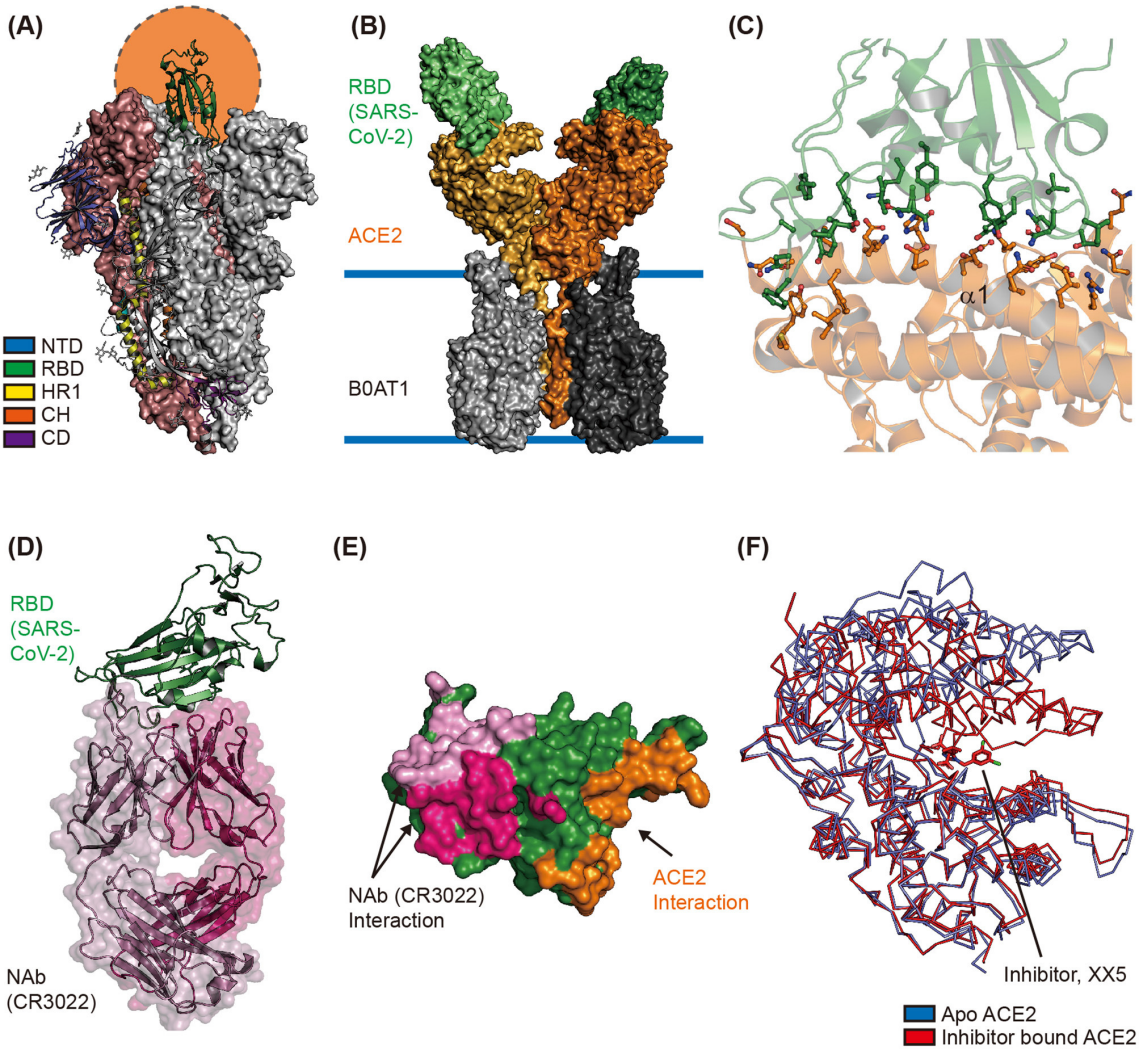
Structural characterization of the interface between ACE2 and SARS-CoV-2. **(A)** Overall structure of the spike glycoprotein (S) of SARS-CoV-2 in its homotrimeric conformation. One up and open conformation of the trimer is shown; the up position of the receptor binding domain (RBD), shown in green, is indicated by the orange circle (PDB ID 6VXX). The N-terminal domain (NTD), RBD, HR1, CH, and C-terminal domain (CD) are shown in blue, green, yellow, orange, and purple, respectively. **(B)** The CryoEM structure of human ACE2 in complex with the RBD of SARS-CoV-2 and B0AT1 (PDB ID 6M17). The overall structure reveals that human ACE2 forms a homodimer (orange and light-yellow) with B0AT1 (dark and light gray), which is located in the transmembrane region. The two SARS-CoV-2 RBDs are shown as dark and light green surfaces. **(C)** The interaction interface between RBD and ACE2 is shown (PDB ID 6M0J). The residues involved in the interaction between SARS-CoV-2 RBD and hACE2 are represented with stick models in green and orange, respectively. Alpha helix 1 (α1) of hACE2 is also labeled. **(D)** The overall structure of SARS-CoV-2 RBD in complex with its neutralizing antibody CR3022 (PDB ID 6W41). The Fab regions of the heavy and light chains are shown in hot pink and pink, respectively. SARS-CoV-2 RBD is shown in green. **(E)** Structural comparison of interfaces between SARS-CoV-2 RBD and Nab or hACE2. The interaction interfaces with the light chain of CR3022, heavy chain of CR3022, and hACE2 are shown in pink, hot pink, and orange, respectively. **(F)** Hinge movement of hACE2 upon binding of the enzyme inhibitor. The Apo form (PDB ID 1R42) and inhibitor-bound form (PDB ID 1R4L) are superimposed and shown in blue and red, respectively.

The cryo-electron microscopy (CryoEM) structure of the extracellular domain of the S protein of SARS-CoV-2 revealed a homotrimeric conformation (Wrapp et al., 2020). The binding of RBD—located in the S1 subunit—to hACE2 on the host cell surface initiates interaction between the virus and the host cell; therefore, the switching conformation of RBD is considered an important event for viral entry (Shang et al., 2020). CryoEM studies revealed that the RBD in two out of three S proteins binds to the N-terminal domain (NTD) of the neighboring protomer of the S protein. These inter-molecular interactions result in a down (closed) conformation, wherein the hACE2 interaction interfaces are buried inside the structure. Moreover, the RBD in the third S protein forms an up (open) conformation to facilitate binding with the N-terminal region of hACE2 (Figure 2A) (Wrapp et al., 2020). The cryoEM study of SARS-CoV-2 S showed that single RBD formed an open conformation in an asymmetric trimer. The structural comparisons between the S protein of SARS-CoV (PDB ID 6CRZ) and SARS-CoV2 (PDB ID 6VSB) showed that the major structural differences came from RBD in a closed conformation. Although the RBD of S from SARS-CoV and SARS-CoV-2 were largely resembled, the SARS-CoV-2 RBD showed a higher binding affinity toward hACE2 than SARS-CoV RBD (Lan et al., 2020; Shang et al., 2020). The CyroEM structure of full-length hACE2 revealed a homodimeric conformation, with each monomer of hACE2 binding to one RBD of the SARS-CoV-2 S protein (Figure 2B) (Yan et al., 2020). The crystal structure of hACE2 in complex with SARS-CoV-2 RBD (PDB ID 6M0J and 6VW1) showed that SARS-CoV-2 RBD binds to the N-terminal region of hACE2 via S19, Q24, T27, F28, D30, K31, H34, E35, E37, D38, Y41, Q42, L45, L79, M82, Y83, Q325, N330, K353, D355, and R357 residues of hACE2 and K417, V445, G446, Y449, Y453, L455, F456, Y473, A475, G476, E484, F486, N487, Y489, Q493, G496, Q489, T500, N501, G502, V503, and Y505 residues of SARS-CoV-2 RBD (Figure 2C) (Shang et al., 2020; Wrapp et al., 2020). Most of these interactions are mediated by α1 of hACE2 (Figure 2C); moreover, an N-glycosylation chain at N90 of hACE2 interacts with SARS-CoV-2 S protein (Shang et al., 2020).

As mentioned earlier, the S1/S2 junction and S2′ site of the S protein are cleaved by furin and TMPRSS2, to enable efficient entry of SARS-CoV-2 into the host cell (Figure 2A). In addition to trypsin, cathepsin L, and elastase, TMPRSS2 is known to activate the S protein and induce virus-cell membrane fusion (Matsuyama et al., 2010). A recent study reported that TMPRSS2 is also essential for SARS-CoV-2 entry into target cells (Hoffmann et al., 2020; Matsuyama et al., 2020).

Accordingly, targeting proteins that participate in SARS-CoV-2 entry can be a potential therapeutic strategy. The use of neutralizing antibodies (NAbs) against SARS-CoV-2's S protein is thought to be promising for the treatment of patients with COVID-19 (Pinto et al., 2020). A Nab—CR3022—known to target SARS-CoV RBD and prevent lung pathology, can also bind to SARS-CoV-2 RBD (ter Meulen et al., 2006; Tian et al., 2020). The crystal structure of SARS-CoV-2 RBD in complex with CR3022 revealed that CR3022 forms a distinct interaction interface with SARS-CoV-2 RBD, and does not overlap with the interaction interface between hACE2 and SARS-CoV-2 RBD (Figures 2D,E). Although CR3022 binds to SARS-CoV RBD and SARS-CoV-2 RBD with binding affinities (Kd) of 1 and 115 nM, respectively, it is unable to neutralize SARS-CoV-2 *in vitro* largely due to its inability to form the interaction interface and its low binding affinity (Pinto et al., 2020; Yuan et al., 2020). However, continuous efforts are being undertaken to identify potent NAbs by collecting plasma from infected individuals, and this has shown significant progress. The P2B-2F6 from SARS-CoV2 infected patients have overlapping residues, G446 and Y449, with higher RBD binding affinity than ACE2/RBD (5.14 and 4.70 nM respectively) (Ju et al., 2020). Furthermore, the interaction interface of C105/RBD overlapped with the ACE2 binding region, and B38 share similar binding structures with prominent neutralizing effects (Barnes et al., 2020; Wu et al., 2020). Also they showed recent concern of mutation in S (D614G) that might increase SARS-CoV-2's transmission rate and has a rare chance to affect the RBD-binding Mab C105, because of the distance between the RBD region and D614 (Barnes et al., 2020). In addition to identifying NAbs targeting SARS-CoV-2's S protein, a pilot trial to use recombinant soluble human ACE2 in COVID-19 patients has been initiated (clinicaltrial.gov #NCT04287686). However, this trial was recently withdrawn as it was not approved by the Center for Drug Evaluation (CDE). Because ACE2 can counter the activation of renin–angiotensin–aldosterone system (RAAS) treatment with ACE2 inhibitors, it can increase ACE2 expression in some patients to compensate for the blocked ACE2 activity (Vaduganathan et al., 2020). In some animal studies, treatment of RAAS inhibitor resulted in increased expression of ACE2 in specific tissues (Ferrario et al., 2005; Soler et al., 2009). In this regard, some researchers hypothesized that treatment of the RAAS inhibitor might enhance the accessibility of SARS-CoV-2 into cells and therefore increase the risk of severity in patients carrying COVID-19 (Fang et al., 2020; Watkins, 2020). However, a recent case population study showed that there was no correlation between use of RAAS inhibitors and increased risk of COVID-19 (de Abajo et al., 2020). The Ramipril, ACE inhibitor showed cardiac protective effects without increased expression of ACE2 (Burchill et al., 2012). These contradictory results suggested that clinical validations of RAAS inhibitors are needed to demonstrate its effectiveness toward COVD-19. The high-resolution X-ray crystal structure of apo-hACE2 and hACE2 in complex with its enzymatic inhibitor MLN-4760 showed that inhibitor binding at the active site of hACE2 can cause large hinge-bending movement (Towler et al., 2004) (Figure 2F). Furthermore, a structure-based drug discovery study showed that an enzymatic hACE2 inhibitor can prevent SARS-CoV infection (Huentelman et al., 2004). Therefore, hACE2 inhibitors can potentially prevent SARS-CoV-2 infection.

Although the structure of human TMPRSS2 is not available yet, homology modeling and *in silico* docking studies have demonstrated the molecular mechanisms of camostat mesylate, nafamostat, and bromhexine hydrochloride in inhibiting TMPRSS2 (Sonawane et al., 2020). In this respect, active site-specific inhibitors of TMPRSS2 can be used as potential antiviral agents against SARS-CoV-2.

### Viral Proteases

The crystal structure of SARS-CoV M^pro^–a cysteine protease—consists of domains 1–3. The catalytic processes of M^pro^ are mediated by the non-canonical Cys-His catalytic dyad located between domains I and II (Anand et al., 2002, 2003). The M^pro^ protein is highly conserved among SARS-CoV, MERS-CoV, and SARS-CoV-2, and it shares the common substrate recognition sequence consisting of LQ(S,A,G) (Ziebuhr et al., 2000; Hegyi and Ziebuhr, 2002; Dai et al., 2020). Among them, the Gln in P1 of the substrate is an important common feature required for their catalytic activity. Human proteases with a similar substrate specificity to that of M^pro^ do not exist; therefore, development of M^pro^ inhibitors is a potential therapeutic strategy for targeting SARS-CoV-2.

**Figure 3:**
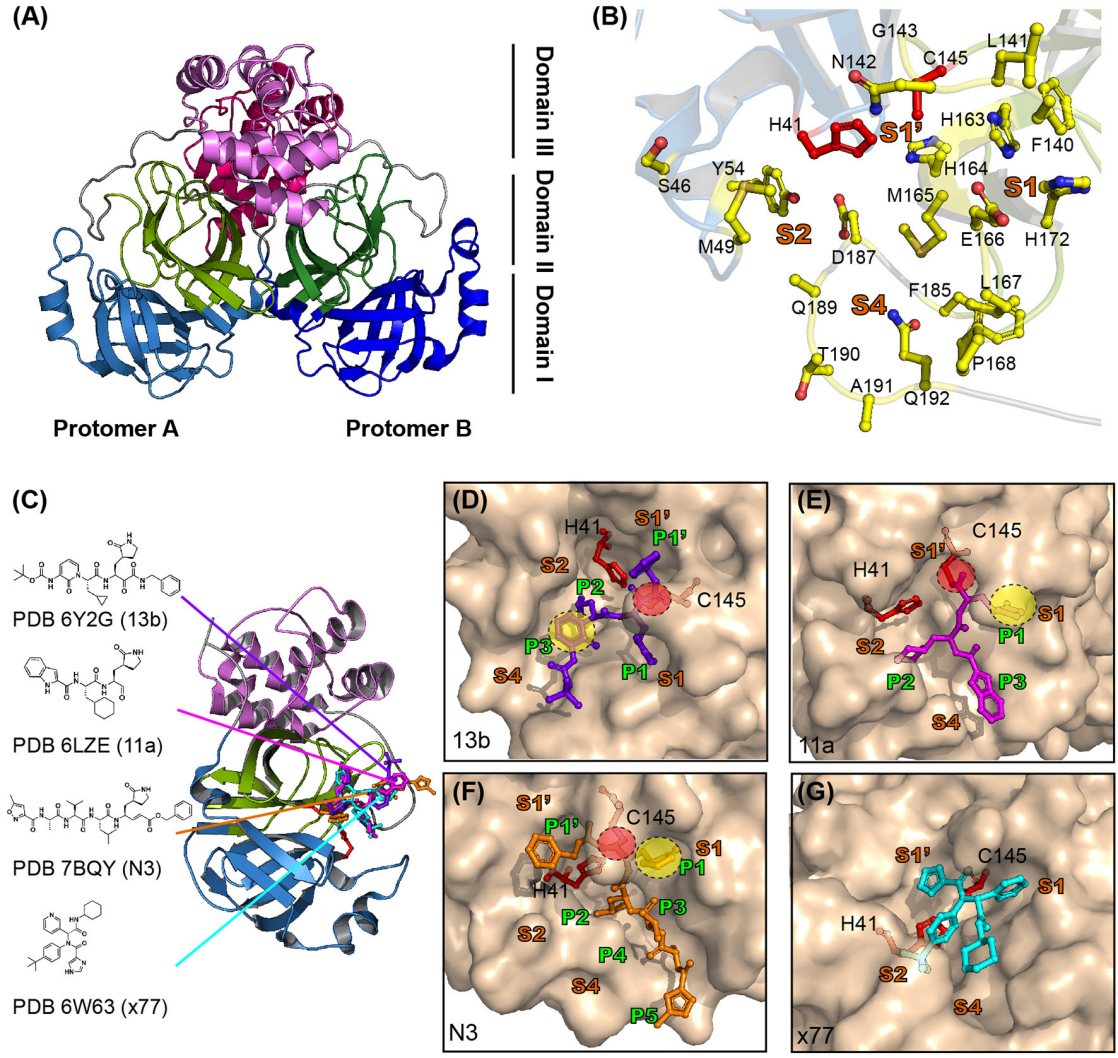
Structure of SARS-CoV-2 viral M^pro^ and its complex with inhibitors. **(A)** The crystal structure of SARS-CoV-2 M^pro^. M^pro^ is a cysteine protease that consists of three domains and two protomers. Protomer *B* is shown in darker colors than protomer *A* and each domain is shown in different colors (sky blue, split pea, and violet represent domains 1, 2, and 3, respectively). **(B)** Substrate binding site of SARS-CoV-2 M^pro^. The substrate binding site of M^pro^ is subdivided into S1, S1′, S2, and S4 (shown in bold orange). The inhibitors bind to 17 residues shown as yellow sticks (H41, S46, M49, Y56, F140, L141, N142, C145, H164, M165, E166, L167, H172, Q189, F185, T190, and Q192). The cysteine-histidine dyad (C145-H41) between domains 1 and 2 is shown in red. **(C)** SARS-CoV-2 M^pro^ in complex with its inhibitors. The structures of SARS-CoV-2 M^pro^ in complex with 13b (PDB ID 6Y2G, purple sticks), 11a (PDB ID 6LZ2, magenta sticks), N3 (PDB ID 7BQY, orange sticks), and x77 (PDB ID 6W63, cyan sticks) are shown. The molecular interaction of each inhibitor in the active site is shown as a surface and stick complex (**D–G** are 13b, 11a, N3, and x77). The γ-lactam ring that plays an important inhibitory role is shown in the yellow circle, and C-S covalent bonds with Cys145 are shown in the red circle.

SARS-CoV-2 M^pro^ consists of three domains, analogous to that of M^pro^ from other CoVs (Figure 3A) (Dai et al., 2020; Jin et al., 2020; Zhang et al., 2020b). The crystal structure of M^pro^ revealed that it forms homodimers (dimeric protomer) through interactions between domain II of protomer A and N-terminal residues of protomer B (Figure 3A) (Zhang et al., 2020b). Homodimerization of M^pro^ is required for its enzymatic activity. Mutational studies on the dimeric interface, as well as crystal structure analysis, revealed that the interaction between two protomers is required to form the S1 pocket at the substrate binding site (Figure 3B) (Anand et al., 2002; Lim et al., 2014; Zhang et al., 2020b). The substrate binding site of SARS-CoV-2 consists of S1′-S1-S2-S4 pockets lined with, H41, S46, M49, Y54, F140, L141, N142, G143, C145, H163, H164, M165, E166, L167, H172, F185, D187, Q189, T190, A191, and Q192 residues (Figure 3B) (Dai et al., 2020; Jin et al., 2020; Zhang et al., 2020b). Notably, the S2 pocket of CoVs is typically hydrophobic and can accommodate the bulky P2 fragment (Figure 3B). Several structure-based drug discovery studies have investigated the interaction of inhibitors in the substrate-binding pockets of SARS-CoV-2 M^pro^ (Figure 3C) (Dai et al., 2020; Jin et al., 2020; Zhang et al., 2020b). A previous study for developing broad spectrum inhibitors targeting CoV M^pro^ showed that inhibitors of SARS-CoV-2 contain a (S)-γ-lactam ring at P1 position to mimic glutamine and occupy the S1 pocket of SARS-CoV-2 M^pro^ (Zhang et al., 2020a). A total of 103 structures of SARS-CoV-2 M^pro^ in both apo and inhibitor complex forms are available in the protein data bank (PDB) database (https://www.rcsb.org/) until 27 April 2020. Zhang et al. (2020b) have developed peptidomimetic α-ketoamide inhibitors targeting SARS-CoV-2 M^pro^. They also solved the crystal structure of M^pro^ in complex with α-ketoamide 13b (PDB ID 6Y2G) and showed the presence of a γ-lactam ring at P1 position and cyclopropyl at P2 position (Figure 3D). The biochemical IC_50_ of SARS-CoV-2, SARS-CoV, and MERS-CoV M^pro^ were found to be 0.67, 0.90, and 0.58 μM, respectively (Zhang et al., 2020b). Simultaneously, Dai et al. (2020) developed inhibitors with an aldehyde-substituted compound at warhead for occupying the S1 site and thus it covalently bonds with the catalytic cysteine of SARS-CoV-2 M^pro^ (PDB ID 6LZE and 6MOK) (Dai et al., 2020) (Figure 3E). These compounds showed high inhibition activity with IC_50_ of 53 and 40 nM *in vitro* and reduced SARS-CoV-2 infection with EC_50_ of 0.53 and 0.72 μM in plaque reduction assay (Dai et al., 2020). The crystal structure of SARS-CoV-2 M^pro^ in complex with the inhibitor compound N3 (PDB ID 7BQY), previously designed to inhibit CoV M^pro^, revealed that N3 occupies the substrate binding pocket and forms a covalent bond with catalytic C145 of SARS-CoV-2 M^pro^. Consistently, the lactam ring at P1 position of N3 forms a hydrogen bond with H163 of SARS-CoV-2 M^pro^ (Figure 3F) (Yang et al., 2005; Jin et al., 2020). X77, a potential inhibitor of SARS-CoV-2 M^pro^, also occupies the substrate binding pocket; however, it does not form covalent bonds (PDB ID 6W63) (Figure 3G).

In conclusion, M^pro^ of SARS-CoV-2 is a key protein that participates in the proteolytic processing of polyproteins and shows no overlapping substrate specificity with any of the known human proteases. Several potent inhibitors share common structural features, including covalent bond formation with catalytic cysteine and a lactam ring at P1 position. Because most inhibitors occupy the substrate binding pocket of SARS-CoV-2 M^pro^, targeting this pocket could be an efficient and safe strategy in terms of toxicity.

### Viral Replication

**Figure 4:**
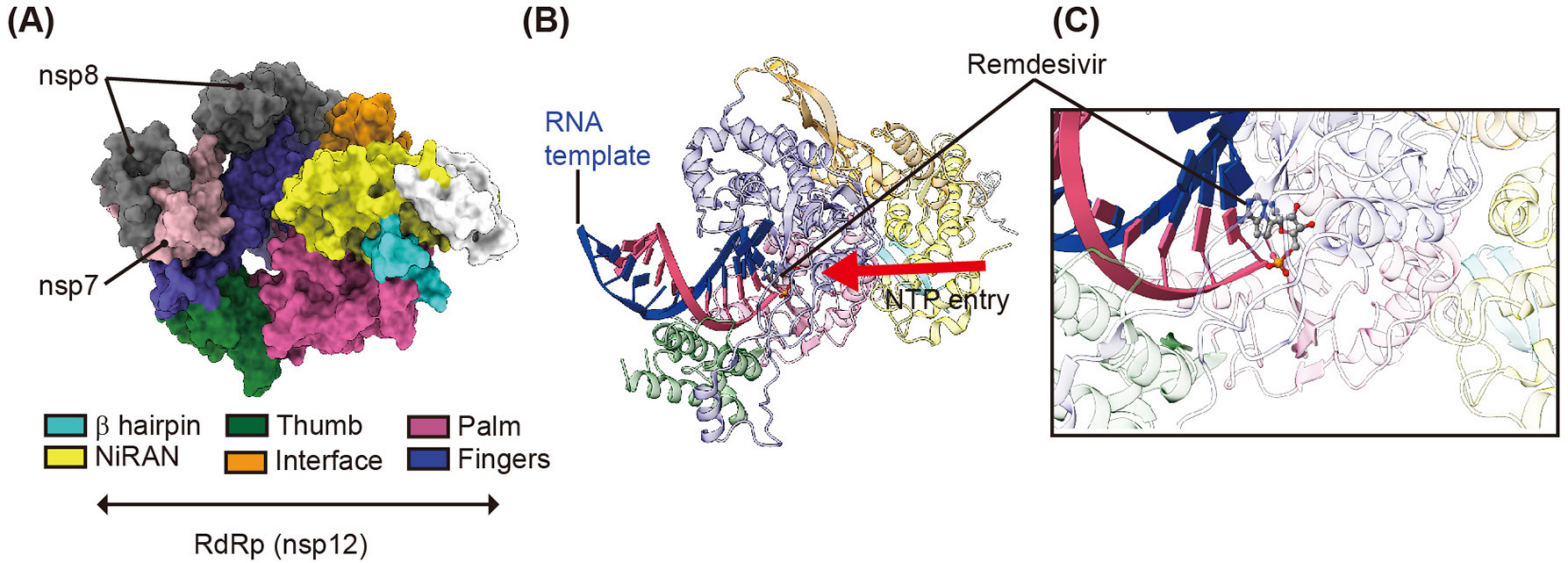
CryoEM structure of RdRp in complex with cofactors (nsp7 and nsp8), RNA template, and remdesivir. **(A)** Surface representation of the CryoEM structure of SARS-CoV-2 RdRp in complex with its cofactors (two nsp8 and one nsp7) (PDB ID 6M71). nsp7 and nsp8 are shown in gray and pink, respectively. The β-hairpin, NiRAN, interface, thumb, palm, and finger of SARS-CoV-2 RdRp are shown in cyan, yellow, green, orange, purple, and blue, respectively. **(B)** A cartoon representation of the overall structure of SARS-CoV-2 RdRp in complex with the RNA template and its inhibitor remdesivir (PDB ID 7BV2). The RNA template and primer strand are shown in blue and red, respectively. The red arrow indicated the direction of NTP entry. **(C)** magnified view of remdesivir monophosphate binding region. Remdesivir covalently binds to the primer RNA strand and interacts with the template RNA.

Replication of SARS-CoV-2 genomic RNA is mediated by a multiprotein complex consisting of several non-structural proteins, such as nsp7, nsp8, nsp12, and nsp14. The functional core of this multiprotein complex consists of RNA-dependent RNA polymerase (RdRp, also called nsp12) (Huang J. et al., 2020). SARS-CoV-2 RdRp plays an important role in the replication and transcription of viral genomic RNA (Figure 1) and its catalytic residues are highly conserved among CoVs (Venkataraman et al., 2018; Huang J. et al., 2020). It is because of this that the nucleotide analog remdesivir (GS-5734, Gilead) was treated to target RdRp of MERS-CoV, SARS-CoV, and SARS-CoV-2 (Warren et al., 2016; Holshue et al., 2020; Wang M. et al., 2020). Although the viral RdRp is a core component of viral replication, nsp7 and nsp8 are still required for full-fill transcriptional activity of RdRp (Zhai et al., 2005; Venkataraman et al., 2018; Kirchdoerfer and Ward, 2019; Gao et al., 2020). The cryoEM structure of nsp12 revealed an N-terminal β-hairpin (aa 31–50), extended nidovirus RdRp-associated nucleotidyl-transferase domain (NiRAN, aa 117–250), interface domain (aa 251–365), and RdRp domain (aa 366–920) consisting of finger, palm, and thumb subdomains (Gao et al., 2020; Yin et al., 2020) (Figure 4A). Structural studies have demonstrated that nsp12 can recognize the RNA template in a sequence-independent manner, suggesting that the enzymatic activity of RdRp is largely sequence independent. The cryoEM structure of SARS-CoV-2 RdRp in complex with an RNA template or its small molecule inhibitor, remdesivir, (Figure 4B) revealed the molecular inhibitory mechanism of remdesivir (Yin et al., 2020). Remdesivir monophosphate interacts with the primer strand and uridine of the template strand by base stacking and hydrogen bonding, respectively, at the center of the catalytic active site of RdRp (Yin et al., 2020) (Figure 4C). The covalent incorporation of remdesivir monophosphate into the primer strand blocks the entry of nucleotide triphosphates to the active site, and terminates the transcriptional activity of RdRp (Yin et al., 2020) (Figure 4B). Other nucleotide analog compounds such as favipiravir, ribavirin, EIDD-1931, and EIDD-2801 may exhibit a similar mechanism of action as remdesivir to inhibit RdRp with non-obligate RNA chain termination (Elfiky, 2020; Sheahan et al., 2020; Wang Y. et al., 2020). Although the U.S. Food and Drug Administration issued an emergency use authorization for remdesivir on May 1, 2020 for the treatment of suspected or laboratory-confirmed COVID-19 in adults and children hospitalized with severe symptoms, the clinical efficacy of remdesivir against SARS-CoV-2 is not known yet. Moreover, no significant clinical benefits of remdesivir against SARS-CoV-2 were observed in a recent randomized, double-blind, placebo-controlled, multicenter clinical trial (ClinicalTrials.gov, NCT04257656) (Wang Y. et al., 2020).

Taken together, compounds that target SARS-CoV-2 RdRp are largely nucleotide analogs because of their ability to form covalent bonds with the viral template RNA and block the catalytic active site of RdRp.

## Conclusions

Zoonotic coronavirus outbreaks such as COVID-19 can not only affect public health but also have a major impact on societies and the global economy. Therefore, global cooperation among academic institutions, governments, and pharmaceutical companies is necessary to overcome COVID-19.

Despite intensive worldwide efforts undertaken by researchers to contain the spread of SARS-CoV-2, COVID-19 has attained pandemic status. Considering that the development of an effective vaccine and new therapeutics are still in the early stages, repurposing FDA-approved and well-characterized drugs might be a pragmatic approach. Consequently, some of these drugs, such as remdesivir, have been approved for emergency use and some are being tested in clinical trials. In addition, combination treatment might be an approach which could achieve synergistic effects and reduce the risk of drug-resistant mutations.

A few studies have shown that some pre-existing drugs are effective for the treatment of patients with COVID-19. In this review, we described the ongoing therapeutic strategies targeting various components of the SARS-CoV-2 life cycle (Table 1). In addition, we provided structural insights into the mechanism of action of well-characterized drugs targeting the interaction between hACE2 and the spike protein of SARS-CoV-2 for viral entry, as well as M^pro^ and RdRp for viral replication. We believe that structural characterization can aid in developing an effective therapeutic strategy not only against COVID-19 but also other viral outbreaks in the future.

## Author Contributions

GJ and HS conceived, designed, did the literature review, provided, and wrote the manuscript. GY assisted in the preparation and design. DK and Y-CK conceived, designed, assisted in the literature, final review, and co-wrote the manuscript. All authors contributed to the article and approved the submitted version.

## Conflict of Interest

The authors declare that the research was conducted in the absence of any commercial or financial relationships that could be construed as a potential conflict of interest.
